# Supplementary material for: Modeling glioblastoma heterogeneity as a dynamic network of cell states
Source: Mol Syst Biol. 2021 Sep 16;17(9):e10105. doi: 10.15252/msb.202010105 (PMC8444284; doi:10.15252/msb.202010105)
Supplement: Supplementary file 6 — Source Data for Figure 5 [file MSB-17-e10105-s004.zip › Figure5A_sourcedata/GSEA_3071/hallmarks_stateI.GseaPreranked.1623742531165/HALLMARK_OXIDATIVE_PHOSPHORYLATION.html]

Details for gene set HALLMARK\_OXIDATIVE\_PHOSPHORYLATION[GSEA]

|  || Dataset | state13071 |
| Phenotype | NoPhenotypeAvailable |
| Upregulated in class | na\_neg |
| GeneSet | HALLMARK\_OXIDATIVE\_PHOSPHORYLATION |
| Enrichment Score (ES) | -0.2781535 |
| Normalized Enrichment Score (NES) | -1.2553545 |
| Nominal p-value | 0.18926975 |
| FDR q-value | 0.31479922 |
| FWER p-Value | 0.997 |
Table: GSEA Results Summary

  

Fig 1: Enrichment plot: HALLMARK\_OXIDATIVE\_PHOSPHORYLATION      
 Profile of the Running ES Score & Positions of GeneSet Members on the Rank Ordered List

  

| PROBE | GENE SYMBOL | GENE\_TITLE | RANK IN GENE LIST | RANK METRIC SCORE | RUNNING ES | CORE ENRICHMENT || 1 | POR |  |  | 515 | 0.499 | -0.1251 | No |
| 2 | MGST3 |  |  | 586 | 0.460 | -0.1234 | No |
| 3 | ATP6V0E1 |  |  | 788 | 0.386 | -0.1631 | No |
| 4 | NQO2 |  |  | 955 | 0.339 | -0.1950 | No |
| 5 | OAT |  |  | 1129 | 0.302 | -0.2306 | No |
| 6 | ISCU |  |  | 1266 | 0.274 | -0.2569 | No |
| 7 | HADHB |  |  | 1271 | 0.274 | -0.2450 | No |
| 8 | SDHD |  |  | 1299 | 0.269 | -0.2400 | No |
| 9 | IDH2 |  |  | 1324 | 0.264 | -0.2343 | No |
| 10 | NDUFA6 |  |  | 1420 | -0.250 | -0.2499 | No |
| 11 | UQCRQ |  |  | 1464 | -0.254 | -0.2502 | No |
| 12 | FH |  |  | 1552 | -0.262 | -0.2629 | No |
| 13 | UQCRFS1 |  |  | 1576 | -0.265 | -0.2569 | No |
| 14 | NDUFB5 |  |  | 1613 | -0.269 | -0.2545 | No |
| 15 | NDUFAB1 |  |  | 1635 | -0.272 | -0.2476 | No |
| 16 | COX6B1 |  |  | 1673 | -0.277 | -0.2451 | No |
| 17 | VDAC3 |  |  | 1728 | -0.284 | -0.2472 | No |
| 18 | NDUFB1 |  |  | 1836 | -0.298 | -0.2640 | Yes |
| 19 | ACAA1 |  |  | 1862 | -0.300 | -0.2569 | Yes |
| 20 | MRPL15 |  |  | 1864 | -0.300 | -0.2429 | Yes |
| 21 | TOMM22 |  |  | 1973 | -0.315 | -0.2591 | Yes |
| 22 | OPA1 |  |  | 2014 | -0.319 | -0.2555 | Yes |
| 23 | IDH3A |  |  | 2020 | -0.320 | -0.2417 | Yes |
| 24 | TIMM9 |  |  | 2068 | -0.328 | -0.2396 | Yes |
| 25 | MRPS12 |  |  | 2073 | -0.330 | -0.2251 | Yes |
| 26 | ATP5F1D |  |  | 2125 | -0.338 | -0.2237 | Yes |
| 27 | PHB2 |  |  | 2129 | -0.339 | -0.2084 | Yes |
| 28 | TIMM8B |  |  | 2139 | -0.341 | -0.1948 | Yes |
| 29 | NDUFB4 |  |  | 2164 | -0.346 | -0.1853 | Yes |
| 30 | CYC1 |  |  | 2208 | -0.354 | -0.1808 | Yes |
| 31 | TIMM50 |  |  | 2255 | -0.364 | -0.1768 | Yes |
| 32 | TIMM10 |  |  | 2296 | -0.372 | -0.1707 | Yes |
| 33 | ATP6V0C |  |  | 2360 | -0.386 | -0.1705 | Yes |
| 34 | TIMM13 |  |  | 2451 | -0.401 | -0.1774 | Yes |
| 35 | MAOB |  |  | 2491 | -0.411 | -0.1691 | Yes |
| 36 | ECI1 |  |  | 2500 | -0.414 | -0.1517 | Yes |
| 37 | VDAC2 |  |  | 2535 | -0.424 | -0.1413 | Yes |
| 38 | LDHB |  |  | 2597 | -0.438 | -0.1381 | Yes |
| 39 | UQCR11 |  |  | 2614 | -0.443 | -0.1216 | Yes |
| 40 | BAX |  |  | 2660 | -0.457 | -0.1128 | Yes |
| 41 | ATP5F1E |  |  | 2721 | -0.477 | -0.1074 | Yes |
| 42 | CYCS |  |  | 2727 | -0.479 | -0.0861 | Yes |
| 43 | NDUFA3 |  |  | 2740 | -0.483 | -0.0666 | Yes |
| 44 | SLC25A5 |  |  | 2765 | -0.493 | -0.0500 | Yes |
| 45 | COX17 |  |  | 2786 | -0.498 | -0.0321 | Yes |
| 46 | COX8A |  |  | 2819 | -0.509 | -0.0171 | Yes |
| 47 | COX6C |  |  | 2825 | -0.511 | 0.0058 | Yes |
| 48 | NDUFA7 |  |  | 2988 | -0.593 | -0.0128 | Yes |
| 49 | ATP1B1 |  |  | 3093 | -0.669 | -0.0110 | Yes |
| 50 | GPX4 |  |  | 3103 | -0.679 | 0.0188 | Yes |
| 51 | SLC25A4 |  |  | 3316 | -0.953 | 0.0029 | Yes |
| 52 | PDP1 |  |  | 3376 | -1.117 | 0.0390 | Yes |
Table: GSEA details [plain text format]

  

Fig 2: HALLMARK\_OXIDATIVE\_PHOSPHORYLATION: Random ES distribution      
 Gene set null distribution of ES for **HALLMARK\_OXIDATIVE\_PHOSPHORYLATION**
